# Meta-Analysis of Cytokine Gene Polymorphisms and Outcome of Heart Transplantation

**DOI:** 10.1155/2013/387184

**Published:** 2013-08-20

**Authors:** Sasitorn Yongcharoen, Sasivimol Rattanasiri, D. Olga McDaniel, Mark McEvoy, Chukiat Viwatwongkaseam, Piangchan Rojanavipart, Ammarin Thakkinstian

**Affiliations:** ^1^Section for Clinical Epidemiology and Biostatistics, Faculty of Medicine, Ramathibodi Hospital, Mahidol University, Bangkok 10400, Thailand; ^2^Department of Surgery, The University of Mississippi Medical Center, Jackson, MS 39216, USA; ^3^Centre for Clinical Epidemiology and Biostatistics, The University of Newcastle, Newcastle, NSW 2300, Australia; ^4^Department of Biostatistics, Faculty of Public Health, Mahidol University, Bangkok 10400, Thailand

## Abstract

We performed a systematic review and meta-analysis with the aim of assessing the association between cytokine gene polymorphisms and graft rejection in heart transplantation. We identified relevant studies from Medline and Embase using PubMed and Ovid search engines, respectively. Allele frequencies and allele and genotypic effects were pooled. Heterogeneity and publication bias were explored. Four to 5 studies were included in pooling of 3 gene polymorphisms. The prevalences of the minor alleles for TNF**α**-308, TGF**β**1-c10, and TGF**β**1-c25 were 0.166 (95% CI: 0.129, 0.203), 0.413 (95% CI: 0.363, 0.462), and 0.082 (95% CI: 0.054, 0.111) in the control groups, respectively. Carrying the A allele for the TNF**α**-308 had 18% (95% CI of OR: 0.46, 3.01) increased risk, but this was not significant for developing graft rejection than the G allele. Conversely, carrying the minor alleles for both TGF**β**1-c10 and c25 had nonsignificantly lower odds of graft rejection than major alleles, with the pooled ORs of 0.87 (95% CI: 0.65, 1.18) and 0.70 (95% CI: 0.40, 1.23), respectively. There was no evidence of publication bias for all poolings. An updated meta-analysis is required when more studies are published to increase the power of detection for the association between these polymorphisms and allograft rejection.

## 1. Introduction

Heart transplantation is a treatment of choice for the end-stage heart diseases with the goal to improve patient survival and the quality of life [1, 2]. Acute or chronic graft rejection is an unwanted outcome of transplantation, which occurs approximately 40% during the first six months after allograft transplantation [3]. Many factors have been reported influencing graft rejection, including patient characteristics, quality of the graft, HLA compatibility, environmental factors, immunosuppressant regimen, and genetic predispositions. Identification of reliable molecular markers that allows accurate prediction of clinical outcomes before a rejection episode occurs may help better management of the patients at higher risk for rejection.

 Cytokine gene polymorphisms are responsible for the regulation of inflammatory response and are known to play a role in mediating allograft rejection after transplantation. Transforming growth factor-beta 1 (TGF*β*1) and tumor necrosis factor alpha (TNF*α*) are among the key cytokines which have been reported in association with inflammation and rejection episodes after renal [4] and liver transplantations [5]. The involvement of polymorphisms includes variations in the genes coding for TGF*β*1 at codon 10 (position +869 (T → C)) and codon 25 (position +915 (G → C)) [6–10] and for TNF*α* at position −308 (G → A) [8–12]. The TGF*β*1 gene is located on chromosome 19q13.1 (MIM#190180), and the polymorphisms have variable effects on cytokine production. The TNF*α* gene is located on chromosome 6p21.3 (MIM#191160), and such polymorphisms have generated a high responder genotype associated with acute liver allograft rejection [5]. 

 These polymorphisms had been reported in association with poor clinical outcomes after heart transplantation. However, gene effects identified in these studies were controversial; that is, some studies found positive associations between polymorphisms and rejection episodes [3, 6–8, 13–15] while others did not [10, 12, 16]. The discrepancy might result from variations in the study populations and thus different gene frequencies, or alternatively due to poor power of detection for negative findings. We therefore conducted a systematic review and applied meta-analysis to increase the power of detection for the association between these polymorphisms and allograft rejection.

## 2. Materials and Methods

### 2.1. Search Strategy

Relevant studies were identified from MEDLINE and Embase since initiations to February 2013 using PubMed and Ovid search engines, respectively. The search strategies for PubMed were “(heart failure OR graft failure OR graft rejection) AND ((TGF beta OR TGF-beta OR transforming growth factor beta) OR (TNF alpha 308 OR TNF alpha-308 OR tumor necrosis factor alpha)) AND (heart transplantation OR myocardial transplantation) AND (Gene OR allele OR polymorphism).” Searching was limited to English and human studies.

### 2.2. Selection of Study

Identified studies were independently selected by two reviewers (Sasitorn Yongcharoen and Sasivimol Rattanasiri) based on previously designed inclusion and exclusion criteria. Studies were selected regardless of ethnicity if they met the following criteria: pediatric or adult patients with heart transplantation, assessment of at least one of the following gene effects: TGF*β*1 at codon 10 or codon 25, or TNF*α*-308, and had acute or chronic graft rejection as the outcome of interest. Studies with insufficient data for pooling, that is, no frequencies of genotypes or alleles for each polymorphism and outcomes, were excluded.

### 2.3. Outcome of Interest

The outcome of interest was acute or chronic graft rejection, which was defined according to original studies. Briefly, a severe graft rejection was defined as histopathological finding for rejection scores of grade 3A or higher according to already established criteria by the International Society of Heart and Lung Transplantation classification [17].

### 2.4. Risk of Bias Assessment

The quality of studies was also independently assessed by the same 2 reviewers (Sasitorn Yongcharoen and Sasivimol Rattanasiri) based on a risk of bias score for genetic association studies [18]. The score was divided into 5 domains, including information bias (ascertainment of outcome and gene), confounding bias, selective reporting of outcomes, population stratification, and Hardy-Weinberg equilibrium (HWE) assessment in the control group. Each question was checked whether there was low risk of bias, and the low risk was graded as yes and the high risk was graded as no. If there was insufficient information or the information was not mentioned, it was graded as “unclear” or “not assessable.” Disagreement between the two reviewers was solved by a senior reviewer (Ammarin Thakkinstian). 

### 2.5. Statistical Analysis

We used data in the control group to estimate a pooled allelic prevalence. Hardy-Weinberg equilibrium (HWE) was assessed [19], and only studies that observed HWE were included in pooled analyses [20, 21]. The odds ratio (OR) of minor versus major allele effects on graft rejection along with 95% confidence interval (CI) was estimated. Heterogeneity of allele effects across studies was checked using a *Q* test and the degree of heterogeneity was quantified by *I* ^2^. If heterogeneity was present (i.e., the *Q* test was significant or *I* ^2^ > 25%), the OR was pooled using the DerSimonian and Laird method; otherwise the inverse-variance method was used.

 For genotype analysis, heterogeneity was assessed for OR_1_ (minor homozygous versus major homozygous genotypes) and OR_2_ (heterozygous versus major homozygous genotypes) using the same methods as per-allele analysis. A mixed logistic regression was applied by fitting graft rejection on genotypic variables, treating study as a random effect and genotype as a fixed effect [22–24]. A likelihood ratio (LR) test was used to assess the overall gene effects. All analyses were performed using STATA version 12. A *P* value less than 0.05 was considered statistically significant, except for the heterogeneity test in which *P*-value < 0.10 was used.

## 3. Results

### 3.1. Identifying Relevant Studies

**Figure 1:**
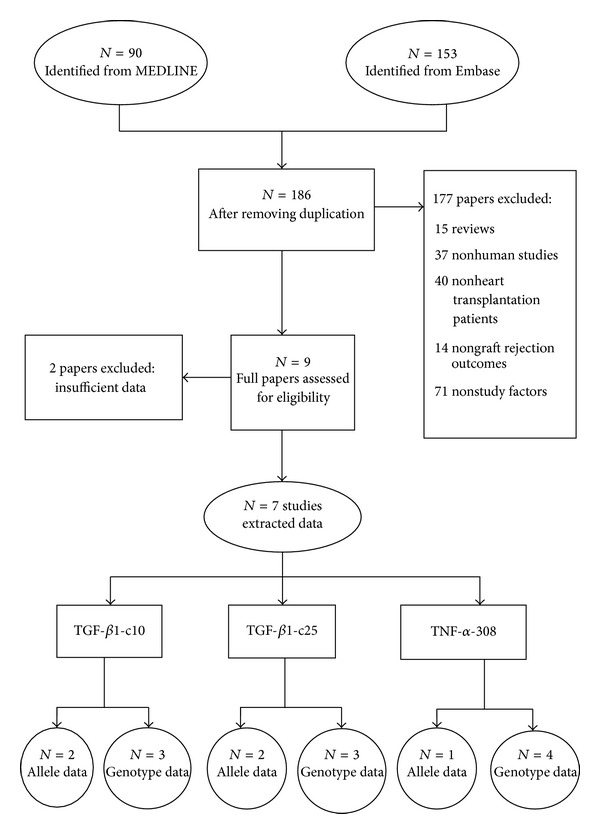
Flow of study selection.

The flow of identification and selection of studies in the meta-analysis is described in Figure 1. Ninety and 153 studies were identified from Medline and Embase, respectively. After duplicates were removed, 186 titles or abstracts were screened, of which 177 studies were ineligible leaving 9 remaining studies. Two studies were excluded due to insufficient data leaving 7 studies for further data extractions. A study by McDaniel et al. [9] had insufficient data and author had provided additional data, in which the number of subjects was larger than reported. The characteristic of these 7 studies has been described in Table 1. Among them, 5 selected studies were conducted in North America, and the rest were in the United Kingdom and Europe. The mean age ranged from 5.3 to 7.5 years and 45.6 to 52.0 years in pediatrics and adults, respectively. The percentage of males ranged from 56.8 to 83.1 percent. All studies were cohort designs. 

### 3.2. Risk of Bias Assessment

The results of bias assessment of the 7 studies have been presented in Table 2. All studies had low risk of bias from ascertainment of graft and nongraft rejections and selective outcome reports. Most studies (85.7%) did not adjust for potential confounding effects nor assessed the HWE. Therefore, there were high risks of bias from confounding bias and noncompliance with the HWE. The genotyping methods were clearly described in 5 (71.4%) studies, so bias due to genotyping error might be low for these studies. Population stratification was unclear and was not accessible from most of these studies (85.7%), so bias from population stratification might be present. 

### 3.3. TNF*α*-308

Five studies [8–12] reported association between TNF*α*-308 polymorphism and graft rejection (*n* = 659). The G and A allele frequencies were described according to studies and have been presented in supplement Table 1 see Supplementary Material available online at http://dx.doi.org/10.1155/2013/387184. All except one study [12] did not observe HWE and thus, these were not included in further poolings. The pooled prevalences of the A allele were 0.169 (95% CI: 0.092, 0.246) and 0.166 (95% CI: 0.129, 0.203) in rejection and nonrejection groups, respectively. The ORs for A versus G alleles were highly heterogeneous (*Q* = 11.10, d.f. = 3, *P* = 0.011, and *I* ^2^ = 73.0%) across the studies. The pooled ORs with DerSimonian and Laid method were 1.18 (95% CI: 0.46, 3.01), suggesting that individuals carrying A allele had 18% increased risk for developing graft rejection than those carrying G allele. However, this risk was not statistically significant. The Egger test did not suggest any evidence of publication bias (coefficient = 4.98, SE = 19.04, and *P* = 0.818). 

**Figure 2:**
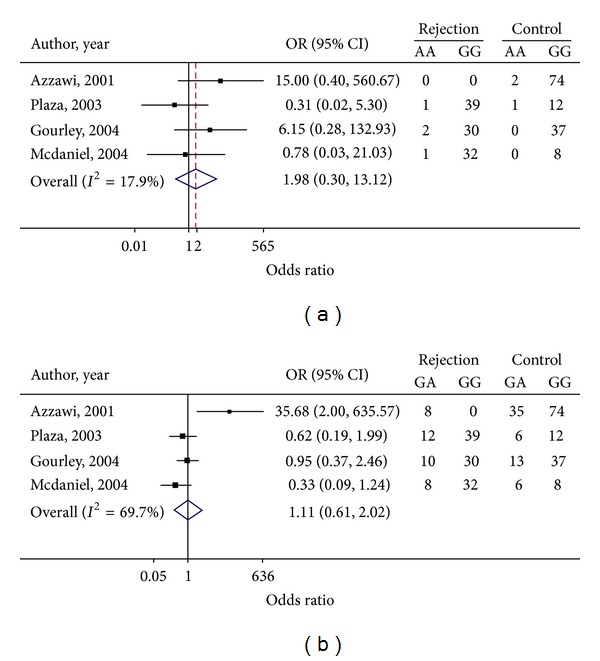
Forest plots of genotypic ORs for TNF*α*-308 polymorphism: (a) TNF*α*-308 AA versus GG and (b) TNF*α*-308 GA versus GG.

 Genotype frequency and estimated OR for each study have been presented in Figure 2. The OR_1_ for AA versus GG was homogenous (*Q* = 3.65, d.f. = 3, *P* = 0.301, and *I* ^2^ = 17.9%) whereas the OR_2_ for GA versus GG was highly heterogeneous (*Q* = 9.89, d.f. = 3, *P* = 0.020, and *I* ^2^ = 69.7%); see Figures 2(a) and 2(b). Applying the mixed logit regression yielded the pooled OR_1_ and OR_2_ of 1.98 (95% CI: 0.30, 13.12) and 1.11 (95% CI: 0.61, 2.02), respectively, which suggested that individuals carrying AA and GA genotypes had 98% and 11% higher odds of graft rejections than those carrying GG genotype but these were not statistically significant. The Egger test did not suggest any asymmetry for both ORs (coefficient = 7.788, SE = 5.409, and *P* = 0.287 for OR_1_; coefficient = 3.177, SE = 2.337, and *P* = 0.307 for OR_2_). The estimated lambda was 0.42 (95% CI: 0.02, 0.97), suggesting that an additive mode was most likely.

### 3.4. TGF*β*1-c10

Five studies [6–10] assessed association between TGF*β*1-c10 and graft rejection (*n* = 399). Allele frequencies across outcomes were described and all studies observed HWE and have been presented in supplement Table 2. The minor C allele prevalence was 0.382 (95% CI: 0.291, 0.472) in rejection and 0.413 (95% CI: 0.363, 0.462) in nonrejection groups, respectively. The allelic effects for C versus T alleles were homogenous across the studies (*Q* = 2.64, d.f. = 4, *P* = 0.619, and *I* ^2^ = 0%) with the pooled OR of 0.87 (95% CI: 0.65, 1.18), which suggested that individuals carrying C alleles were at 13% lower odds than those carrying T allele. Publication bias was assessed by graphing a funnel plot, which indicated little asymmetry (coefficient = 2.54, SE = 2.29, and *P* = 0.348). 

**Figure 3:**
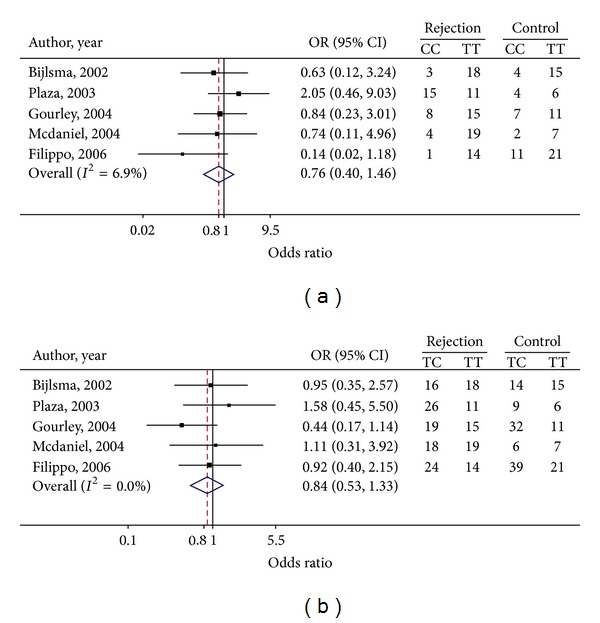
Forest plots of genotypic ORs for TGF*β*1-c10 polymorphism: (a) TGF*β*1-c10 CC versus TT and (b) TGF*β*1-c10 TC versus TT.

 Genotype frequency and estimated ORs for each study have been presented in Figure 3. Both OR_1_ for CC versus TT and OR_2_ for TC versus TT were mildly heterogeneous across the studies (*Q* = 4.30, d.f. = 4, *P* = 0.367, and *I* ^2^ = 6.9% for OR_1_; *Q* = 3.04, d.f. = 4, *P* = 0.552, and *I* ^2^ = 0% for OR_2_). The pooled OR_1_ and OR_2_ were 0.76 (95% CI: 0.40, 1.46) and 0.84 (95% CI: 0.53, 1.33), respectively (see Figures 3(a) and 3(b)). From this it could be interpreted that individuals carrying CC or TC genotypes were at 24% and 16% lower risk of graft rejections than those carrying the TT genotype. However, these genotype effects were not statistically significant. The estimated lambda was 0.42 (95% CI: 0.02, 0.96), suggesting that there was no effect between a dominant, additive, or recessive mode of effect. The Egger tests were performed and suggested no asymmetry of the funnels for OR_1_ (coefficient = −1.87, SE = 2.17, and *P* = 0.451) and OR_2_ (coefficient = 2.54, SE = 1.95, and *P* = 0.283). 

### 3.5. TGF*β*1-c25

Five studies [6–10] assessed the association between TGF*β*1-c25 and graft rejection (*n* = 387). The G/C allele frequencies and estimated OR have been presented in supplement Table 3. The pooled prevalence of C alleles was 0.056 (95% CI: 0.034, 0.078) and 0.082 (95% CI: 0.054, 0.111) in the rejection and the nonrejection groups, respectively. The allelic effects for C versus G alleles were homogeneous across the studies (*Q* = 2.05, d.f. = 4, *P* = 0.727, and *I* ^2^ = 0%) with the pooled OR of 0.70 (95% CI: 0.40, 1.23). This suggested that individuals carrying C alleles had 30% lower risk (but this was not significant) to develop graft rejection than those carrying G alleles. The Egger test did not suggest asymmetry of funnel (coefficient = 0.23, SE = 1.52, and *P* = 0.890). 

**Figure 4:**
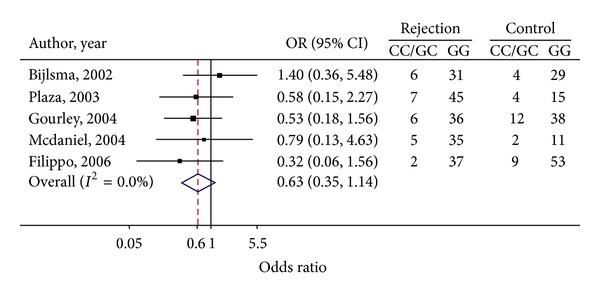
Forest plots of OR for per-genotype effects of TGF*β*1-c25.

 Genotype frequencies and estimated ORs for each study have been presented in Figure 4. Since homozygous CC genotype was very rare, we combined the CC with GC genotypes. The OR for CC/GC versus GG was homogeneous across the studies (*Q* = 2.21, d.f. = 4, *P* = 0.697, and *I* ^2^ = 0.0%) with the pooled OR of 0.63 (95% CI: 0.35, 1.14), shown in Figure 4. This suggested that individuals carrying CC/GC genotype were approximately at 37% lower risk of graft rejection than those with GG genotype, but this did not reach to statistical significance.

## 4. Discussion

We had performed a systematic review and meta-analysis assessing associations between 3 cytokine polymorphisms (i.e., TNF*α*-308, TGF*β*1-c10, and TGF*β*1-c25) and graft rejection in heart transplantation. Four to 5 studies were included in pooling of 3 polymorphisms with a total sample size of 337 to 399 subjects. We observed no significant polymorphism in association with graft rejection. Nonetheless, our results indicated a signal of association between TNF*α*-308 A allele and graft rejection. It was found that individuals carrying A allele would approximately had 18% increased risk of graft rejection relative to those carrying G allele. Conversely for TGF*β*1 at c10 and c25, carry C alleles for both polymorphisms were respectively 13% and 30% lower risk of graft rejection than carry T and G allele.

 Genotypic effects were also estimated for TNF*α*-308 and TGF*β*1-c10 but not for TGF*β*1-c25 due to lack of genotype data. For TNF*α*-308, the estimated OR_1_ for AA versus GG and OR_2_ for GA versus GG were 1.98 and 1.11, respectively, and the estimated lambda was 0.42, suggesting an additive mode of gene effect. However, the 95% confident interval of lambda laid from 0.02 to 0.97, which suggesting that the genetic mode could be a recessive, additive, or dominant effect. This trend of association was similar to previous finding in renal [4] and liver transplantations [5] which also suggested an additive effect of the A allele. These poolings were based on small number of included studies and thus uncertainty of gene effects was still present.

 For TGF*β*1-c10 polymorphism, the genotype effects of CC and TC versus TT were 0.76 and 0.84, respectively. Although the point estimated lambda was closed to the additive effect (lambda = 0.42), this estimation was still uncertain with 95% CI of 0.02 to 0.96. We however could not assess a mode of gene effect for TGF*β*1 at codon 25 polymorphism since there was no CC genotype in non-rejection group for all studies. As for previous report, this polymorphism was in linkage disequilibrium with TGF*β*1 at codon 10 (*r* = 0.30) [4], in which the minor C allele in TGF*β*1 at codon 25 would go with the minor C allele in TGF*β*1 at codon 10. As a result, the mode of gene effect of TGF*β*1 at codon 25 might be similar to the effect of TGF*β*1 at codon 10 polymorphism. However, our finding was in disagreement with the previous finding in renal transplant patients [4]. They found that patients carrying C alleles in both codon 10 and codon 25 were approximately 30% higher risk of graft rejection than those carrying T and G alleles. The inconsistency in the effects might be due to association by chance as for ours or due to small sample size in previous pooling. In addition, linkage disequilibrium of these two polymorphisms might be different direction in different population.

 The strength of our study is multifold. First, we identified all relevant studies which had assessed the association between these polymorphisms and allograft outcomes in heart transplantation. Second, the review was performed based on rigorous analytical methods and thus biases were due to the selection of studies and less likely due to data extraction. Third, data were pooled using both allele and genotype approaches. The allele approach is better than the genotype approach if a minor genotype is very rare in most included studies. The sample size of the allele pooling is doubled and thus increased the power of detection of the gene effect [25]. However, if data of a minor genotype is available in most included studies, pooling using a genotype-approach is better because this method provides the effects of heterozygous and homozygous genotypes, which will lead to suggestions for a mode of gene effect. However, we had limitations. Only small numbers of studies were included in our pooling. Thus, we were still faced with lack of power for detection of gene effects. The estimated post hoc power of test was 78% for the OR (AA versus GG) for TNF*α*-308 and we needed a sample size of 454 to detect this association. Further updated meta-analysis is required if there are more studies published in the literatures. We pooled gene effects on graft rejection based on summary data which were provided from individual studies. Although most studies had considered acute graft rejection, few studies had mixed acute and chronic graft rejections. Among the acute graft rejection, the severity of graft rejection might also be varied; for instance, 6 out of 8 patients died within 3 months after transplant in the study by Azzawi et al. [11]. Recategorizing the outcome should be a more appropriate method and should lead to valid pooling results. However, this required an individual patient data, which is much more time consuming and takes a larger effort than performing a summary data meta-analysis [4]. 

## 5. Conclusion

In summary, this meta-analysis has demonstrated that individuals carrying a minor A allele of TNF*α*-308 polymorphism might have more risk of developing graft rejection, whereas individuals carrying minor allele C alleles for TGF*β*1-c10 and TGF*β*1-c25 polymorphisms might have less susceptibility to develop graft rejections in heart transplantation. Further studies with larger sample sizes are needed to update and confirm the role of these polymorphisms in association with allograft rejection in heart transplantation.

## Supplementary Material

Supplementary Table 1: This table described major and minor allele frequencies of TNFa-308 polymorphism between rejection and controls groups. Prevalence of the major and minor alleles were estimated and pooled across studies. HWE was checked and OR of allele effect was estimated for each study.
Supplementary Table 2: Numbers of major and minor alleles for TGFb1-c10 between groups were described for each study. Prevalence of allele, HWE, and allele effect were reported.
Supplementary Table 3: Numbers of major and minor alleles for TGFb1-c25 between groups were described for each study. Prevalence of allele, HWE, and allele effect were reported.

## Figures and Tables

**Table 1:** Characteristic of studies which were included in meta-analysis.

Author, year	No. of subjects	Country	Design	Mean age	Percent male	Immunosuppressive agents	HLA	Outcome
Azzawi et al. 2001 [11]	119	United Kingdom	Cohort			Cyclosporine (4–12 mg/kg/day) Azathioprine (1-2 mg/kg/day) Prednisolone (0.1–0.2 mg/kg/day)	Not based on HLA matching	AGR/CGR
Bijlsma et al. 2002 [6]	70	The Netherlands	Cohort					AGR
Plaza et al. 2003 [10]	71	Columbia	Cohort	45.6	83.1	Cyclosporine, prednisolone, and azathioprine	Complete match	
Gourley et al. 2004 [8]	92	Pennsylvania	Cohort	52.0	79.0	Cyclosporine, prednisone, and azathioprine/mycophenolate mofetil	87% had >3 mismatch	AGR
McDaniel et al. 2004 [9]	55*	Mississippi	Cohort	—	76.0			AGR
Filippo et al. 2006 [7]	111	Pittsburgh	Cohort	7.5	56.8	Tacrolimus/cyclosporine, and corticosteroid	—	AGR/CGR
Girnita et al. 2008 [12]	322	Washington	Cohort	5.3	57.0	—	—	AGR/CGR

AGR: acute graft rejection; CGR: chronic graft rejection.

*Authors provided data.

**Table 2:** Risk of bias assessment.

Author, year	Ascertainment of GR	Ascertainment of non-GR	Genotyping controls	Population stratification	Confounding bias	Selective outcome reports	HWE
Azzawi et al. 2001 [11]	Yes	Yes	Unclear	NA	No	Yes	No
Bijlsma et al. 2002 [6]	Yes	Yes	Yes	NA	No	Yes	No
Plaza et al. 2003 [10]	Yes	Yes	Yes	NA	No	Yes	Yes
Gourley et al. 2004 [8]	Yes	Yes	Yes	Unclear	No	Yes	No
McDaniel et al. 2004 [9]	Yes	Yes	Yes	Unclear	No	Yes	No
Filippo et al. 2006 [7]	Yes	Yes	Yes	Unclear	No	Yes	No
Girnita et al. 2008 [12]	Yes	Yes	NA	Yes	Yes	Yes	No

NA: not available.
