# Supplementary material for: Meta-Analysis of Cytokine Gene Polymorphisms and Outcome of Heart Transplantation
Source: Biomed Res Int. 2013 Aug 20;2013:387184. doi: 10.1155/2013/387184 (PMC3762075; doi:10.1155/2013/387184)
Supplement: Supplementary file 1 — Supplementary Table 1: This table described major and minor allele frequencies of TNFa-308 polymorphism between rejection and controls groups. Prevalence of the major and minor alleles were estimated and pooled across studies. HWE was checked and OR of allele effect was estimated for each study. Supplementary Table 2: Numbers of major and minor alleles for TGFb1-c10 between groups were described for each study. Prevalence of allele, HWE, and allele effect were reported. Supplementary Table 3: Numbers of major and minor alleles for TGFb1-c25 between groups were described for each study. Prevalence of allele, HWE, and allele effect were reported. [file 387184.f1.docx]

SUPPLEMENT TABLE 1. Allele Frequencies for TNFa-308, Estimated Pooled Prevalence and Odds Ratio of Minor Alleles

| Author, year | Rejection | | | | | Control | | | | | A/G | HWE |
| --- | --- | --- | --- | --- | --- | --- | --- | --- | --- | --- | --- | --- |
| (Reference no.) | No. of | G allele | | A allele | | No. of | G allele | | A allele | | OR (95% CI) | P value |
|  | alleles | Freq. | Prevalence | Freq. | Prevalence | alleles | Freq. | Prevalence | Freq. | Prevalence |  |  |
| Azzawi, 2001 [11] | 16 | 8 | 0.500 | 8 | 0.500 | 222 | 183 | 0.824 | 39 | 0.176 | 4.69 (1.66, 13.26) | 0.517 |
| Plaza, 2003 [10] | 104 | 90 | 0.865 | 14 | 0.135 | 38 | 30 | 0.789 | 8 | 0.211 | 0.58 (0.22, 1.53) | 1.000 |
| Gourley, 2004 [8] | 84 | 70 | 0.833 | 14 | 0.167 | 100 | 87 | 0.870 | 13 | 0.130 | 1.34 (0.59, 3.03) | 0.580 |
| Mcdaniel, 2004 [9] | 82 | 72 | 0.878 | 10 | 0.122 | 28 | 22 | 0.786 | 6 | 0.214 | 0.51 (0.17, 1.56) | 1.000 |
| Girnita, 2008* [12] | 288 | 251 | 0.872 | 37 | 0.128 | 356 | 310 | 0.871 | 46 | 0.129 | 0.99 (0.63, 1.58) | 0.048 |
| Pooled | 574 | 0.831 (0.754, 0.908) | | 0.169 (0.092, 0.246) | | 744 | 0.834 (0.797, 0.871) | | 0.166 (0.129, 0.203) | | 1.18 (0.46, 3.01) |  |

*Did not comply with HWE rule

SUPPLEMENT TABLE 2. Allele Frequencies for TGFb1-c10, Estimated Pooled Prevalence and Odds Ratio of Minor Alleles

| Author, year | Rejection | | | | | Control | | | | | C/T | | HWE | |
| --- | --- | --- | --- | --- | --- | --- | --- | --- | --- | --- | --- | --- | --- | --- |
| (Reference no.) | No. of | C allele | | T allele | | No. of | C allele | | T allele | | OR (95% CI) | | P value | |
|  | alleles | Freq. | Prevalence | Freq. | Prevalence | alleles | Freq. | Prevalence | Freq. | Prevalence |  | |  | |
| Bijlsma, 2002 [6] | 74 | 22 | 0.297 | 52 | 0.703 | 66 | 22 | 0.333 | 44 | 0.667 | 0.85 (0.41, 1.73) | | 1.000 | |
| Plaza, 2003 [10] | 104 | 56 | 0.538 | 48 | 0.462 | 38 | 17 | 0.447 | 21 | 0.553 | 1.44 (0.68, 3.04) | | 1.000 | |
| Gourley, 2004 [8] | 84 | 35 | 0.417 | 49 | 0.583 | 100 | 46 | 0.460 | 54 | 0.540 | 0.84 (0.47, 1.51) | | 0.085 | |
| Mcdaniel, 2004 [9] | 82 | 26 | 0.317 | 56 | 0.683 | 30 | 10 | 0.333 | 20 | 0.667 | 0.93 (0.38, 2.26) | | 1.000 | |
| Filippo, 2006 [7] | 78 | 26 | 0.333 | 52 | 0.667 | 142 | 61 | 0.430 | 81 | 0.570 | 0.66 (0.37, 1.18) | | 0.466 | |
| Pooled | 422 | 0.382 (0.291, 0.472) | | 0.618 (0.528, 0.709) | | 376 | 0.413 (0.363, 0.462) | | 0.587 (0.538, 0.637) | | | 0.87 (0.65, 1.18) | |  |

SUPPLEMENT TABLE 3. Allele Frequencies for TGFb1-c25, Estimated Pooled Prevalence and Odds Ratio of Minor Alleles

| Author, year | Rejection | | | | | Control | | | | | C/G | HWE |
| --- | --- | --- | --- | --- | --- | --- | --- | --- | --- | --- | --- | --- |
| (Reference no.) | No. of | C allele | | G allele | | No. of | C allele | | G allele | | OR (95% CI) | P value |
|  | alleles | Freq. | Prevalence | Freq. | Prevalence | alleles | Freq. | Prevalence | Freq. | Prevalence |  |  |
| Bijlsma, 2002 [6] | 74 | 6 | 0.081 | 68 | 0.919 | 66 | 4 | 0.061 | 62 | 0.939 | 1.37 (0.37, 5.07) | 1.000 |
| Plaza, 2003 [10] | 104 | 7 | 0.067 | 97 | 0.933 | 38 | 4 | 0.105 | 34 | 0.895 | 0.61 (0.17, 2.23) | 1.000 |
| Gourley, 2004 [8] | 84 | 7 | 0.083 | 77 | 0.917 | 100 | 12 | 0.120 | 88 | 0.880 | 0.67 (0.25, 1.78) | 1.000 |
| Mcdaniel, 2004 [9] | 80 | 6 | 0.075 | 74 | 0.925 | 26 | 2 | 0.077 | 24 | 0.923 | 0.97 (0.18, 5.14) | 1.000 |
| Filippo, 2006 [7] | 78 | 2 | 0.026 | 76 | 0.974 | 124 | 9 | 0.073 | 115 | 0.927 | 0.34 (0.07, 1.60) | 1.000 |
| Pooled | 420 | 0.056 (0.034, 0.078) | | 0.944 (0.922, 0.966) | | 354 | 0.082 (0.054, 0.111) | | 0.918 (0.889, 0.946) | | 0.70 (0.40, 1.23) |  |
